# Genito-Urinary and Syphilitic Diseases

**Published:** 1899-06

**Authors:** Byron H. Daggett

**Affiliations:** Buffalo, N. Y.


### GENITO-URINARY AND SYPHILITIC DISEASES.

Conducted by BYRON H. DAGGETT, M. D., Buffalo, N. Y.

SYPHILITIC HEMIPLEGIA.

HAROLD N. MAYER in a clinical lecture (*Chicago Clinic, Nov.,* 1898,) states that syphilitic poison much more frequently causes thrombosis and cerebral softening than rupture of a blood-vessel. Dana states that less than one-sixth of cases of cerebral hemorrhage are due to lues. The patient, male, 37 years old, printer, contracted syphilis 9 years before and apparently recovered. About five years after the infection, was attacked with sudden giddiness and a sinking sensation, followed for three days by mental confusion. About six months afterward he suffered another attack, less severe, but causing a slight catch in his right arm, which continued for four weeks. Six months later he forgot himself for a short time. Two and one-half years ago he suffered another attack of dizziness which apparently passed off, but was followed during the day by twitching of the right arm and leg and the following morning he awakened with complete paralysis of the right side of the face, right arm and leg. After this attack of paralysis the syphilitic nature of the process was recognised and he was given a thorough course of anti-syphilitic treatment. Under this treatment his condition steadily improved so that he was able to partly resume his occupation.

His physical condition is fair; there is a slight defect in his gait, he can use his arm nearly as well as ever, the right side of the face is smoother than the left. His speech and pupils of eyes are normal. Knee jerks are slightly exaggerated. This case illustrates a typical hemiplegic attack of syphilitic origin. Usually syphilitic hemiplegia is due to thrombosis and not hemorrhage. Thrombosis is indicated by the prodromal attacks which precede the complete paralysis, the mode of onset of the attack, the twitching of the muscles and slow development of the paralysis. Dana alleges that almost all cases of hemiplegia occurring during sleep are thrombotic. The early symptoms were important and should have been sufficient to have diverted attention to a possible syphilitic infection within a few years past.

Every syphilitic patient who complains of disturbed consciousness and speech, vertigo, numbness, tingling neuralgic pains should usually have a specific treatment. In this case vigorous specific treatment relieved the hemiplegia and inhibited the return of the symptoms and probably if the correct relation of the luetic infection and the prodromal symptoms had been recognised and appropriate treatment applied, hemiplegia would not have happened. The golden opportunity in the treatment of nervous syphilis is in the beginning and it should be vigorous treatment. Mercury and the iodides should be used to saturation. If benefit does not come early from this treatment suspend it.

### SYPHILITIC ERUPTIONS AND SIGNS IN THE SKIN OF FORMER SYPHILITICS.

G. H. Fox (*N. Y. Med. Jour.*, April 8, 1899,) states that syphilis of the internal organs is more or less obscure, but cutaneous syphilis is marked by characters so distinct and plain that "he who runs may read." It usually presents such striking and pathognomonic features that its nature is apparent to the eye.

The chief characteristics of syphilodermata are the color, absence of itching and peculiarity of configuration. The color sign is not always typical and cannot be relied upon as a basis of diagnosis. The absence of itching is a common rule with a few exceptions. Pustular syphiloides with crust formation frequently produce pruritis.

The most reliable basis for diagnosis and the most characteristic clinical feature of syphilodermata is its peculiar configuration. The eruptions, which appear during the first three months or the first year, are extensively disseminated and perfectly symmetrical. There is a tendency to corybiform arrangement or grouping in certain relapsing eruptions, yet the eruption on one half of the body is usually duplicated upon the other half. The tendency after the first year is asymmetry and limitation to a portion of the body. The late lesions tend to group and form in a circle; to serpiginous or peripheral extension of the patches and to gummatous ulceration. In many cases the disease may run its course and leave no perceptible trace. In doubtful cases the patient should be stripped and carefully examined from head to foot. In no case is a physician justified in certifying that a patient is free from syphilis.

The cutaneous manifestations of previous syphilis, in the absence of late eruption, are either pigmentary or cicatricial. Pigmentation may last for years, especially upon the leg, still it is not reliable as a sign of a previous existing syphilis. Cicatrices may last for a lifetime, yet they are not reliable signs.

There is nothing absolutely characteristic about a single scar left by a syphilitic lesion. The number of scars, their location and peculiar arrangement indicate their origin beyond a doubt. Location of scars is often quite as characteristic as their peculiar conformation. Small, rounded, smooth, white and depressed scars, grouped or semi-circular, located upon inner and upper surfaces of the leg, indicate syphilitic origin. Traumatism and eczema will produce similar scars, lacking this peculiar grouping, location and configuration.

